# Psychological Typhoon Eye Effect During the COVID-19 Outbreak

**DOI:** 10.3389/fpubh.2020.550051

**Published:** 2020-12-08

**Authors:** Guixiang Wang, Yan Zhang, Simiao Xie, Pu Wang, Guanghui Lei, Yueran Bian, Fei Huang, Jingyuan Zhang, Xiaochen Cao, Na Luo, Mingyan Luo, Qiang Xiao

**Affiliations:** ^1^School of Educational Science, Central China Think Tank, Huazhong University of Science and Technology, Wuhan, China; ^2^City College, Wuhan University of Science and Technology, Wuhan, China; ^3^Department of Rehabilitation Medicine in the Seventh Affiliated Hospital, Sun Yat-sen University, Shenzhen, China; ^4^Guangdong Engineering and Technology Research Center for Rehabilitation Medicine and Translation, Guangzhou, China; ^5^Institute of Medical Robots of Shang Hai Jiao Tong University, Shanghai, China; ^6^Center of Student Development Research and Guidance, Huazhong University of Science and Technology, Wuhan, China; ^7^Hospital of Huazhong University of Science and Technology, Wuhan, China

**Keywords:** COVID-19 epidemic, psychological typhoon eye effect, mental health intervention, psychological stress, self-designed questionnaire

## Abstract

**Background:** The COVID-19 outbreak in Wuhan, Hubei, has brought serious consequences to the lives and mental health of people and has induced psychological stress and affected behavior.

**Methods:** This study used self-designed questionnaires and SPSS to analyze the psychological and behavioral responses of people in different regions during the COVID-19 pandemic and to check for the presence of “psychological typhoon eye” (PTE) effects. The questionnaires adopted three measurement subscales, namely, the risk cognitive subscale, stress response subscale, and behavioral response subscale, and these were administered online (www.wjx.cn) to investigate the psychological and behavioral conduct of respondents from three areas that have been affected by COVID-19 to varying degrees. Exploratory factor analysis and principal component analysis were conducted to explore the factorial structure of these subscales, and confirmatory factor analysis was conducted to explore the structural validity of the questionnaires. The analysis results were used to build a revised 18-item questionnaire which validity was evaluated via ANOVA and LSD.

**Results:** Results confirm the presence of PTE in the research areas during the onset of the COVID-19 outbreak and highlight some significant differences in the cognition and emotions of the residents in these areas. PTE affected the cognition, emotions, and cognitive and emotional responses of the respondents but did not affect their behavioral responses.

**Conclusion:** The findings underscore the urgency of providing sustainable mental health care services across different areas during the COVID-19 outbreak. The residents of those areas worst hit by the pandemic, who may not have taken the situation seriously, require emotional guidance the most. Meanwhile, the residents of other areas, who showed the most negative psychological reactions to the pandemic, require a sense of security, a timely “disconnection” from negative information, an accurate cognition of stress, and an acceptance of self-responses.

## Introduction

A new pneumonia infection was reported for the first time in Wuhan, Hubei province, China, at the end of December 2019 (1). On January 12, 2020, the World Health Organization (WHO) temporarily recommended labeling the pneumonia as a new coronavirus: “2019-nCoV acute respiratory disease” (2). On February 8, 2020, the official for China's National Health Committee issued a notice on the temporary naming of the pneumonia as a new coronavirus infection, and the Chinese name for the coronavirus pneumonia was the “new coronavirus pneumonia” or “NCP” (3) when there was no official English name. On February 11, 2020, the novel coronavirus pneumonia was named “COVID-19” by WHO director-general Tan Taisai in Geneva, Switzerland, and the official English name became “COVID-19” (4, 5).

Based on the rapidly increasing number of confirmed and detected cases reported in Wuhan (6), COVID-19 is highly contagious, and it had posed a great threat to the health of the people of China and the world within a very short time (7, 8). The notification of the WHO on December 31, 2019, by the Chinese Health Authorities prompted health authorities in Hong Kong, Macau, and Taiwan to increase border surveillance, and this generated concern and fear that it could mark the emergence of a novel and serious threat to public health (9, 10). The virus was recognized by China and the WHO as a major public health event because of the uncertainty and complexity of its development (4), its ability to cause group behavior and the spread of public negative emotions, and its ability to have a serious impact on people's mental health and affect their normal life, work routines, and social stability (11, 12). As a major catastrophic emergency, COVID-19 also had a broad and lasting influence, attracting the extensive attention of the media and being the subject of comprehensive media broadcasts as its influence has expanded further. Due to a variety of factors, such as environmental change (social development), humans are increasingly susceptible to both natural and technological disasters (13). In addition, with the rapid development of network communications, information cost is cheap and dissemination speed fast, and the masses were easily affected by the network of public opinion, which has led to deepening panic and uneasiness within this epidemic situation (14).

Previous studies have reported two special phenomena of regional perceived risk, namely, the typhoon effect and the ripple effect. The typhoon eye effect (15) indicates that the cognition of risk events at the epicenter is lower than the perceived risk at the surrounding areas, whereas the ripple effect (16, 17) indicates that the impact of risk events spreads out in a circle and gradually declines along with an increasing geographical distance. The psychological typhoon eye (PTE) effect focuses on the feelings and needs of people. After a disaster, those people living close to the center of the event or in high-risk areas are at risk of experiencing the worst consequences, hence triggering a “ripple effect” (18, 19). Zhang et al. (17) revealed an inverted U-shaped relationship between the distance of working adults from the pandemic epicenter and their burnout and found that both typhoon and ripple effects may be observed in the same disaster event.

Maderthaner et al. (20) found that those residents living near nuclear reactors have a lower risk assessment of nuclear reactors than those living farther away. Melber et al. (21) also found that people living within the vicinity of nuclear facilities have a better evaluation of the safety of these facilities than the public. Lima (22) examined the distance between residents and waste incinerators in a 5-year longitudinal study and found that people who are living closer to incinerators have a higher risk perception and show less support for these structures compared with those living farther away. However, over time, these subjects developed a habitual response, that is, their risk perception was reduced.

Based on this phenomenon, Liang and Xue (23) introduced the concept of PTE, which posits that the psychological response of an individual located closer to the center of a disaster is calmer than that of an individual located farther away (24). For instance, the 2003 SARS outbreak in China triggered significant PTE effects where the risk awareness and psychological stress of people during the peak period were lower than those during the off-peak period (15). The same psychological effect was reported by Li et al. (25) after the 2008 Wenchuan earthquake. Meanwhile, Zheng et al. (26) proposed an “involvement” version of PTE and argued that the more they are involved in mining, the less villagers are concerned about pollution risks. Many scholars have also explored the causes of the PTE effect by using cognitive dissonance theory (27), simple exposure effect (21), and individual knowledge and experience theory (28).

Inspired by these theories, this study checks for any differences in the psychological and behavioral responses of people living across different regions during the early stage of the COVID-19 pandemic and determines whether a PTE effect has emerged during this period. This study defines the PTE effect as the spread of psychological and behavioral responses (15) and contends that the behavioral and psychological distress (29, 30) of people living in the worst-hit areas are less severe than those of people living outside these areas (i.e., those people living at the COVID-19 epicenter are the calmest). Given that the residents living outside the worst-hit areas show poorer cognition, emotions, and behaviors than those living at the epicenter, the impact of the COVID-19 pandemic has spread out and gradually increased along with geographical distance, thereby canceling out the ripple effect.

## Methods

### Questionnaire Measurement Procedures

Following environmental psychology research (31) that examines the effects of the environment on individuals especially in the face of danger, this study investigates the PTE effect of the COVID-19 outbreak in Wuhan, Hubei, by using self-designed questionnaires. The pandemic has seriously affected the lives and mental health of people, thereby warranting an examination of their psychological stress and behaviors. We collected data on the risk cognition, stress response, behavioral response, and socio-demographic information [i.e., age, gender, marital status, education level, and physical conditions (i.e., COVID-19 infection)] of individuals living in three areas, namely, Wuhan, the cities around Wuhan in Hubei Province, and the cities outside Hubei, that have been affected by COVID-19 to different degrees. The questionnaire employed three subscales. First, the risk cognitive subscale (RCS) asked the questions “Do you think the NCP is serious now?,” “Do you think that you are in danger in the face of the pandemic?,” “What are your chances of catching the NCP?,” and “Do you think that the NCP can be cured?” The respondents can respond positively or negatively. Second, the stress response subscale (SRS) asked the questions “Have you been afraid of the pandemic for no reason?,” “Do you feel more nervous than usual?,” “Do you feel depressed?,” “Are you under great pressure?,” “Are you losing weight?,” and “Are you becoming sensitive and suspicious?” The respondents can answer in a variety of ways, including calm, tension, anger, fear, bored, worry, and happy, among others. Third, the behavioral response subscale (BRS) asked the questions “Do you pay attention to authoritative information?,” “Do you focus on information related to the pandemic?,” “Are you eager to investigate?,” “Do you talk to strangers?,” and “Do you look for information related to self-adjustment?” (32).

Each question was rated on a 4- or 5-point scale, with 1 denoting “least severe or not consistent” and 4 or 5 denoting “most severe or very consistent.” The scores received by each item were then averaged. For example, the item related to the severity of the epidemic in Wuhan can receive a compound score of 4.33 (32).

Sampling was conducted in three areas between January 29–31, 2020, when the epidemic had been spreading for 1 month. This period fell during the second week after the regional governments adopted policies closing cities on January 23, 2020 (33); people were seriously threatened by the epidemic, and their lives and mental health are severely been affected. The questionnaire was compiled through the Questionnaire Star platform (Wenjuanxing, http://www.wjx.cn), and the distribution and completion of the questionnaire were accomplished using WeChat, QQ, and Sina microblog.

A total of 2,046 residents from the three selected areas completed the questionnaire on 29 January, and exploratory factor analysis (EFA) and principal component analysis (PCA) were conducted to explore the factorial structure of the three subscales. Meanwhile, 2012 residents completed the questionnaire on January 30, and confirmatory factor analysis (CFA) was conducted to explore the structural validity of the subscales.

#### PCA of the Risk Cognitive Subscale (RCS)

The KMO test and Bartlett's test of sphericity (Kaiser–Meyer–Olkin measure of sampling adequacy = 0.62, chi-square = 815.17, df = 15, *p* < 0.001) indicated that the correlation matrices on which the PCA was based were suitable for analysis. An examination of the scree plot indicated that the extracted components could be restricted to two, suggesting a two-factor model with 6-item: cognition of danger and cognition of protection consciousness. Exploratory factor analysis showed that the eigenvalues were greater than one (altogether explaining 49.51% of the variance), and the factor loadings ranged from 0.52 to 0.72, suggesting that the risk cognitive subscale's structural validity was acceptable (see Table 1).

**Table 1:** Items loadings, eigenvalues and variance of the RCS with PCA.

**Factor**	**Item**	**Number**	**Loading**	**Eigenvalue**	**% of variance**
Recognition of danger	The severity of the epidemic	R1	0.62	1.74	25.25
	Necessary isolation of villages	F2	0.64		
Cognition of protection consciousness	Own risk	R2	0.6	1.23	24.26
	Probability of catching the NCP	R3	0.52		
	Protective measures' identification	F1	0.69		
	NCP can be cured	R4	0.72		

#### PCA of the Stress Response Subscale (SRS)

The KMO test and Bartlett's test of sphericity (Kaiser–Meyer–Olkin measure of sampling adequacy = 0.92, chi-square = 10,587.91, df = 55, *p* < 0.001) indicated that the correlation matrices on which the PCA was based were suitable for analysis. An examination of the scree plot indicated that the extracted components could be restricted to two, suggesting a two-factor model with 11 items: emotional responses and somatic reactions. Exploratory factor analysis showed eigenvalues greater than one (explaining a total of 58.27% of the variance) and factor loadings ranging from 0.50 to 0.83, suggesting that the structural validity of the SRS was acceptable (see Table 2).

**Table 2:** Items loadings, eigenvalues and variance of the SRS with PCA.

**Factor**	**Item**	**Number**	**Loading**	**Eigenvalue**	**% of variance**
Emotional responses	More nervous and anxious	Q1	0.73	5.26	35.33
	Afraid for no reason	Q2	0.74		
	Easily upset or frightened	Q3	0.82		
	Feel depressed	Q4	0.77		
	Unable to calm down	Q5	0.83		
Somatic reactions	Losing weight	T1	0.50	1.15	22.94
	Feel tired for no reason	T2	0.62		
	Affecting normal work and rest	Y4	0.74		
	Under great pressure	L1	0.74		
	Getting angry or grumpy	Y1	0.68		
	Becoming sensitive and suspicious	L5	0.67		

#### PCA of the Behavioral Response Subscale (BRS)

The KMO test and Bartlett's test of sphericity (Kaiser–Meyer–Olkin measure of sampling adequacy = 0.84, chi-square = 4,604.52, df =28, *p* < 0.001) indicated that the correlation matrices on which the PCA was based were suitable for analysis. An examination of the scree plot indicated that the extracted components could be restricted to two, suggesting a two-factor model with eight items: attention to information and behavioral reactions. Exploratory factor analysis showed eigenvalues greater than one (altogether explaining 57.22% of the variance) and factor loadings ranging from 0.56 to 0.84, suggesting that the structural validity of the BRS was acceptable (see Table 3).

**Table 3:** Items loadings, eigenvalues and variance of the BRS with PCA.

**Factor**	**Item**	**Number**	**Loading**	**Eigenvalue**	**% of variance**
Attention to information	Attention to authoritative information	G1	0.78	1.25	16.01
	Information of self-adjustment	L4	0.78		
Behavioral reactions	Think all have novel coronavirus	R5	0.84	3.33	41.21
	Washing or cleaning hands	X3	0.77		
	Eager to have an investigation	Y5	0.71		
	Initiative to avoid strangers	J4	0.81		
	Focusing on the epidemic information	X1	0.70		
	Dare not talk to strangers	J3	0.56		

**Figure 1:**
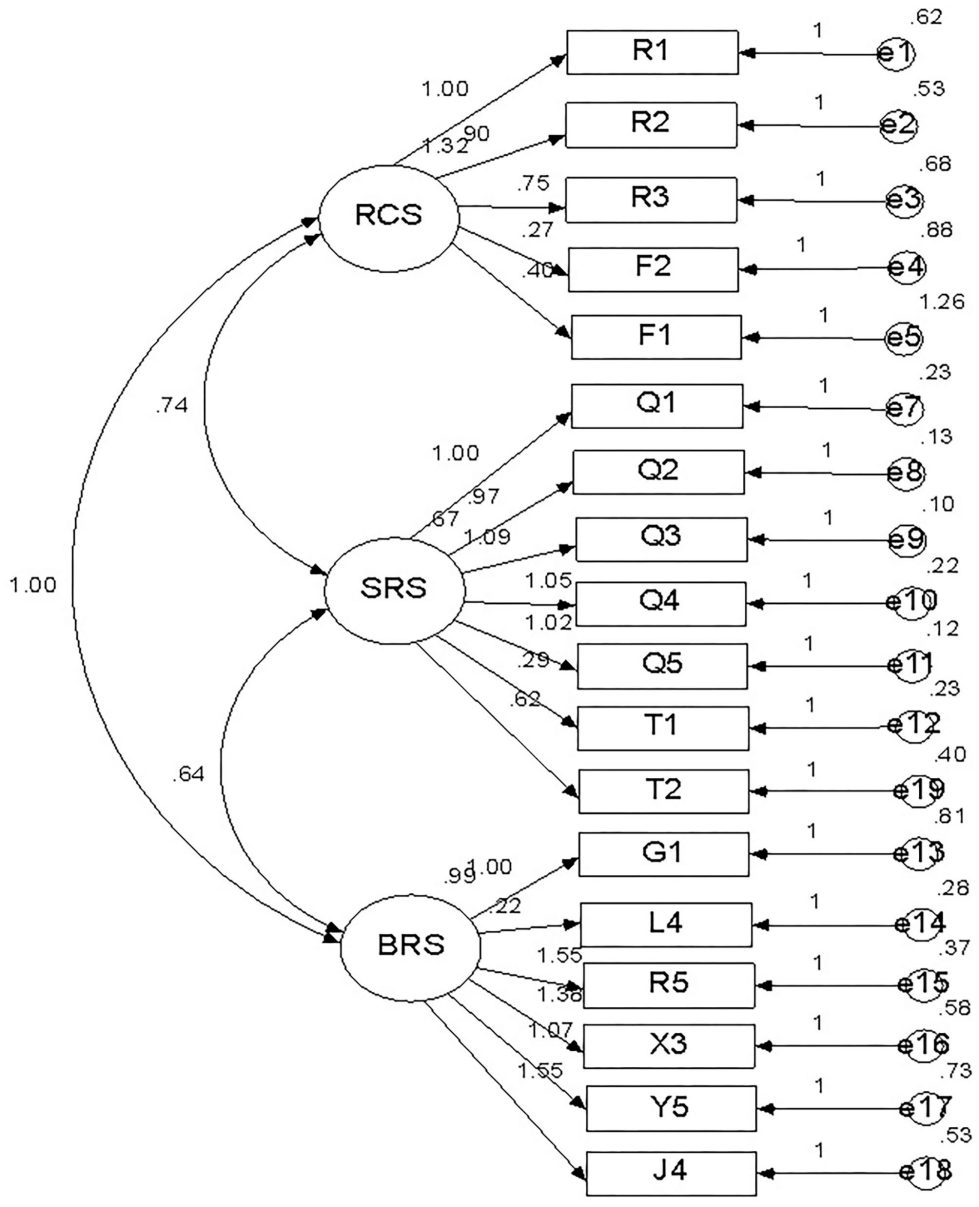
Fit indexes of the 3-subscale model with 18 items.

To further verify the consistency between the model and the real situation, we conducted a confirmatory factor analysis on the data obtained from the formal questionnaire. The fit indexes of the 3-subscale model of 25 items (see Tables 1–3) were not ideal. After deleting the items with low correlations with this factor in the RCS, SRS, and BRS, it can be seen from Table 4 that the fit indexes of the 3-subscale model with 18 items (Figure 1) were higher than those of the 3-subscale model with 25 items and conform to the theoretical concept of this study. The fit indexes of the two models are shown in Table 4.

**Table 4:** Evaluation of questionnaire models.

**Index**	**CMIN/df**	**RESEA**	**NFI**	**IFI**	**CFI**	**GFI**
3-sub-scale model of 24 items	20.92	0.10	0.70	0.72	0.71	0.85
3-sub-scale model of 18 items	25.67	0.11	0.73	0.74	0.74	0.89

Briefly, the questionnaire was divided into three subscales: the risk cognition subscale (RCS), the stress response subscale (SRS), and the behavioral response subscale (BRS). Each subscale had two factors representing different psychological and behavioral states. The RCS consisted of five items, the SRS consisted of seven items, and the BRS consisted of six items. The structure of the RCS, SRS, and BRS are shown below (Table 5).

**Table 5:** The structure of the RCS, SRS, and BRS.

**Factor**	**Recognition of danger**	**Cognition of protection consciousness**
RCS	The severity of the epidemic	Protective measures' identification
	Necessary isolation of villages	Own risk
		Probability of catching the NCP
**Factor**	**Emotional responses**	**Somatic reactions**
SRS	More nervous and anxious	Losing weight
	Afraid for no reason	Feel tired for no reason
	Easily upset or frightened	
	Feel depressed	
	Unable to calm down	
**Factor**	**Attention to information**	**Behavioral responses**
BRS	Attention to authoritative information	Think all have novel coronavirus
	Information of self-adjustment	Washing or cleaning hands
		Eager to have an investigation
		Initiative to avoid strangers

### Formal Investigations

#### Participants

A revised 18-item questionnaire (Table 5) was closed on January 30–31 by 4,076 residents after the exploratory factor analysis. Participants included 1,363 (33.44%) residents of Wuhan, 1,320 (32.38%) residents of cities around Wuhan in Hubei province, and 1,393 (34.18%) residents of cities outside Hubei province. There were 1,929 males and 2,147 females with an average age of 20.17 ± 2.88 years; this was a representative group, generalized due to the homogeneity of the group, and young people were recruited via social media. All respondents had not been infected with the COVID-19 and voluntarily participated in the survey (Table 6).

**Table 6:** Description of samples in three areas.

**Areas**	***N***	**Gender (M/F)**	**Mean age (SD)**
Wuhan City	1,363/1,363	600/763	19.85 (2.80)
Cities around Wuhan in Hubei province	1,320/1,320	630/690	19.52 (1.74)
Cities outside Hubei province	1,393/1,393	699/694	21.11 (3.50)

The variables were generally distributed, and the multiple testing was controlled.

#### Statistical Analyses

The statistical analyses were performed using SPSS 20.0 for Windows (SPSS Inc., Chicago, Illinois). The statistical methods and data analysis results were described in detail as follows.

**Figure 2:**
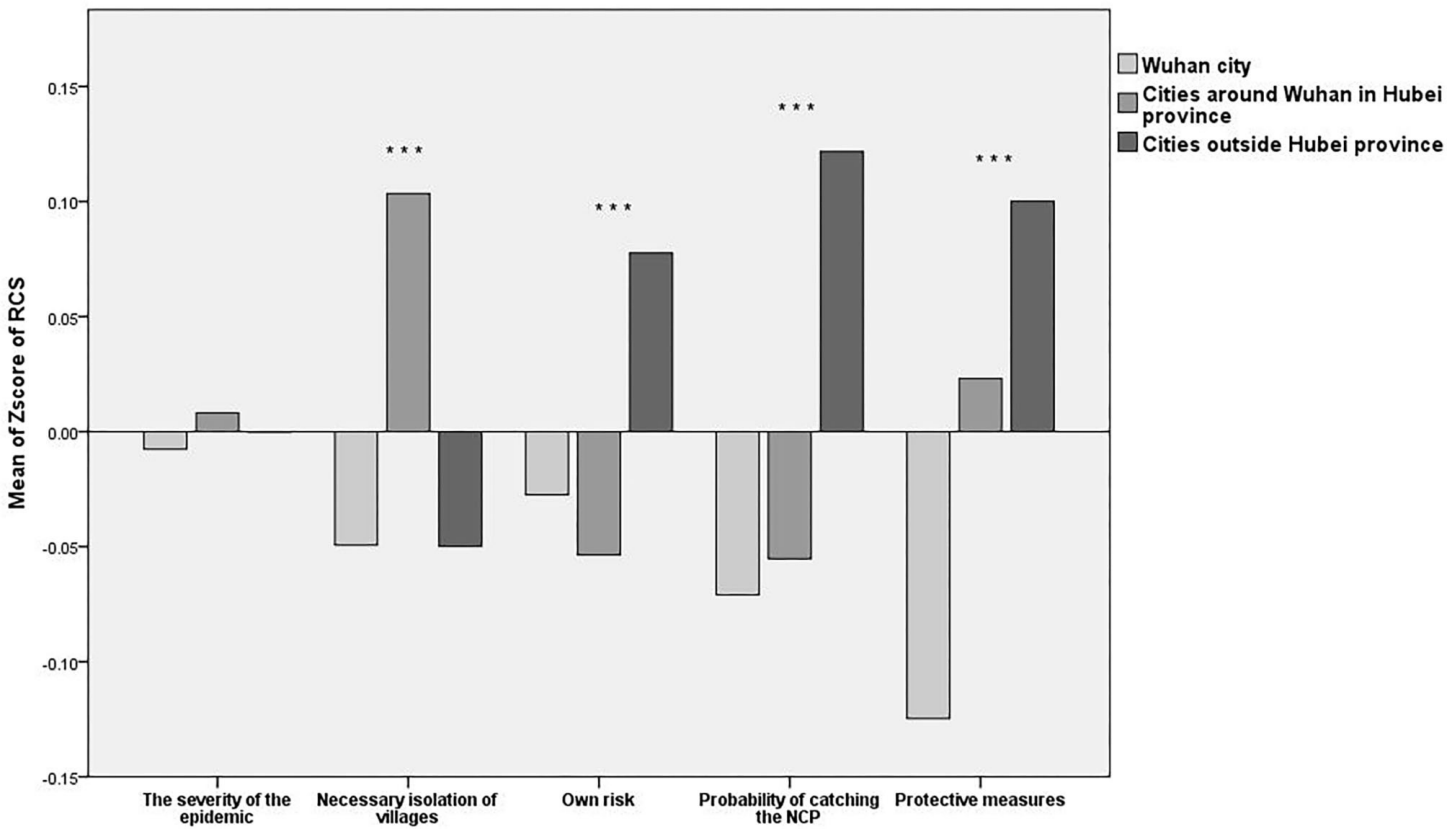
Mean Z-scores of the Risk Cognitive subscale (RCS). ***P < 0.001.

**Figure 3:**
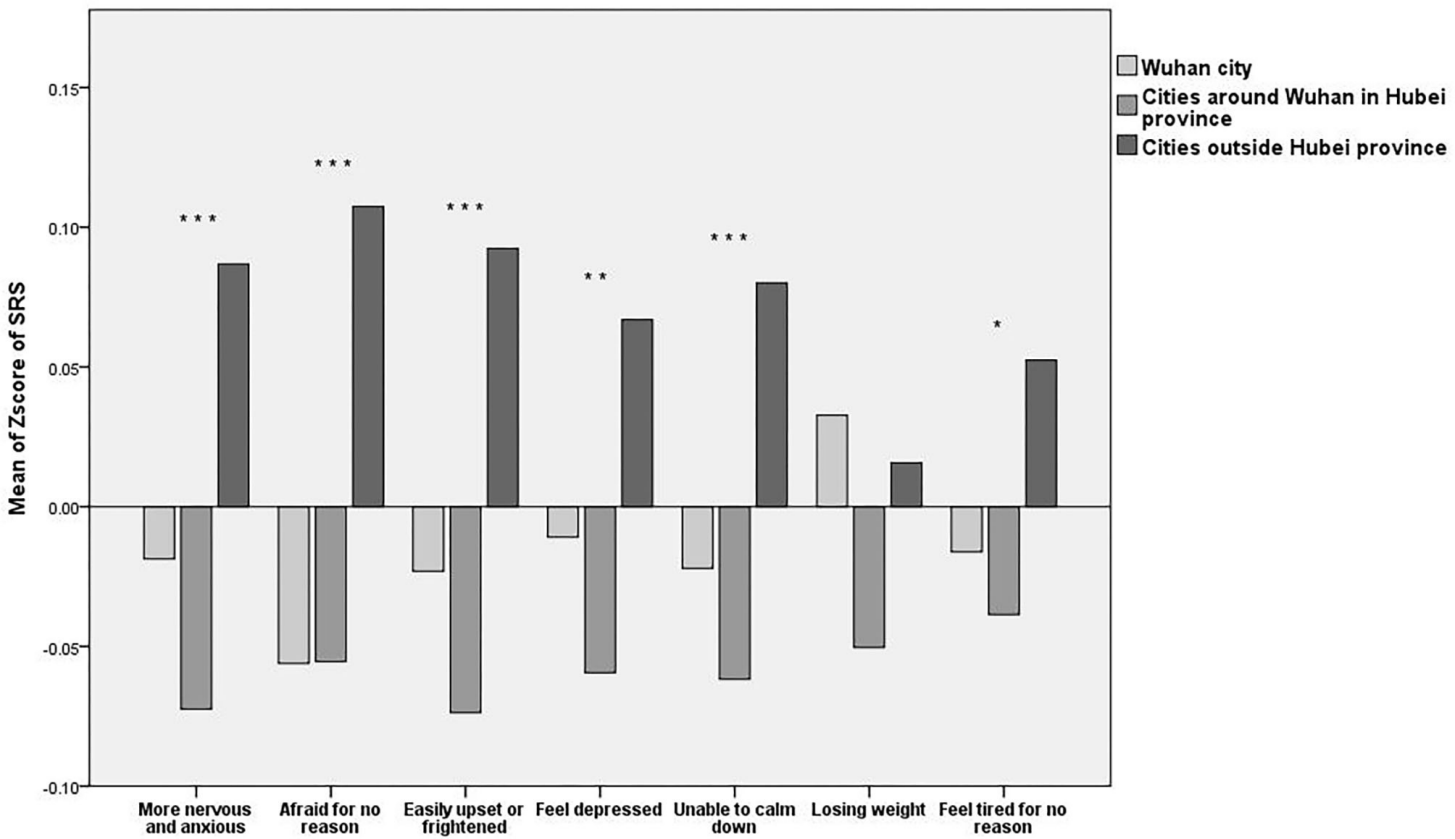
Mean Z-scores of the Stress Response subscale (SRS). *P < 0.05, **P < 0.01, and ***P < 0.001.

**Figure 4:**
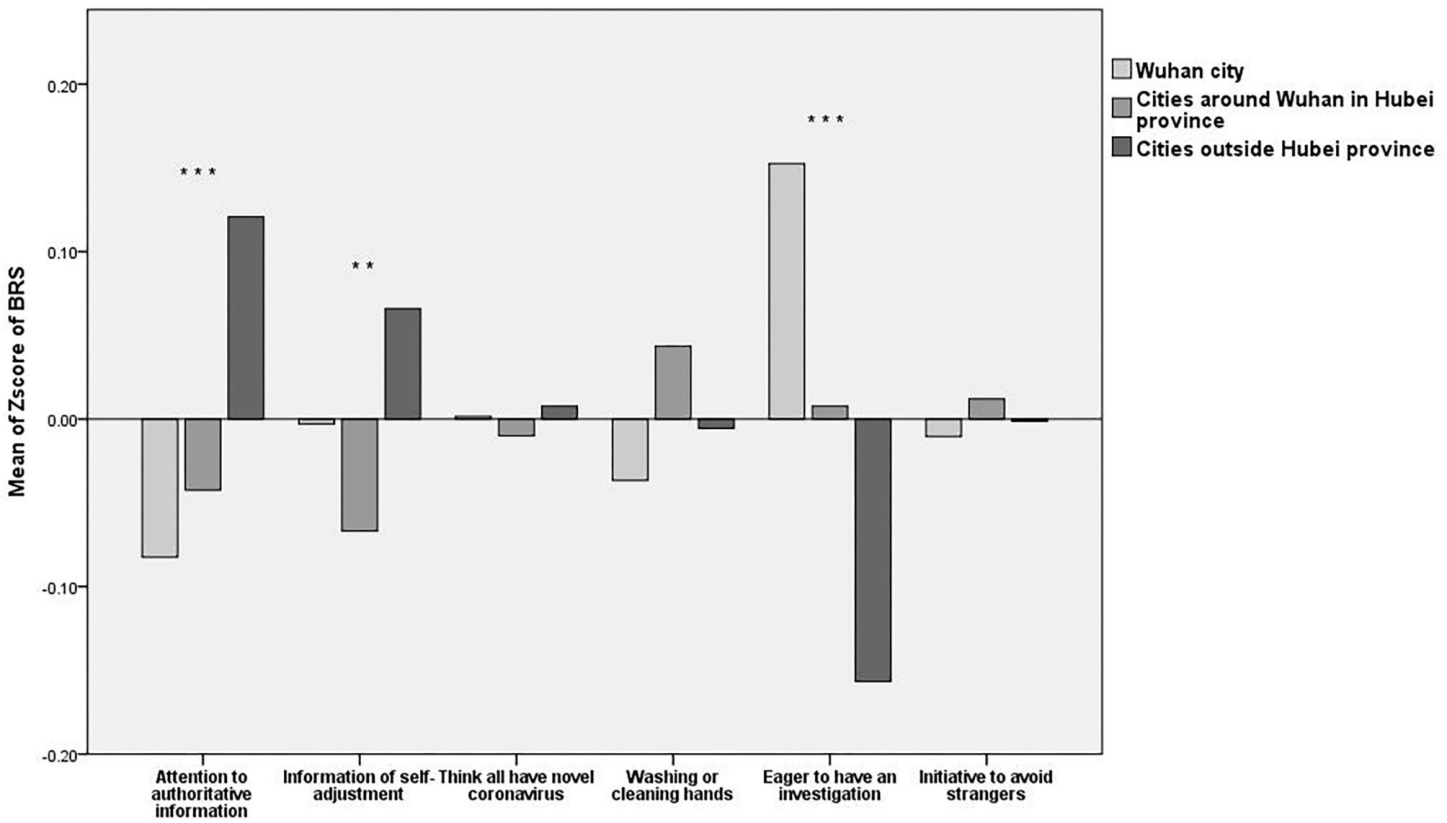
Mean Z-scores of the Behavioral Response subscale (BRS). **P < 0.01, and ***P < 0.001.

We conducted the ANOVA for the items in the three subscales for the three regions and the LSD for the multiple comparisons. The results of the ANOVA and LSD showed that there were statistically significant differences among the three groups with respect to their RCS, SRS, and BRS scores, and there were some commonalities among some items with regard to the three subscales (see Figures 2–**4**).

## Results

### Differences in Risk Cognition Among the Residents of the Three Areas

#### Cognition of Danger

The ANOVA and LSD showed that there were no significant differences in cognition of the epidemic severity [*F*_(2, 4,073)_ = 0.083, *P* = 0.921] among the residents of the three areas. Most of the participants agreed that the epidemic was very serious. However, there was a major difference in terms of the necessary isolation of villages [*F*_(2, 4,073)_ = 10.498, *P* < 0.001] among the residents of the three areas. Residents of cities around Wuhan in Hubei province were the most supportive of social isolation, they had the greatest fear of the virus spreading, and they supported combating the virus through isolation. Conversely, the residents of Wuhan city were the least supportive.

#### Cognition of Protection Consciousness

The ANOVA and LSD showed that there were significant differences in people's own risk cognition [*F*_(2, 4,073)_ = 6.625, *P* = 0.001], the probability of catching the NCP [*F*_(2, 4,073)_ = 15.865, *P* < 0.001], and protective measures' cognition [*F*_(2, 4,073)_ = 18.073, *P* < 0.001] among the residents of the three areas. Residents of cities outside Hubei province thought they were in the greatest danger, but the residents of Wuhan city and of the cities around Wuhan in Hubei province felt less danger. In addition, residents of cities outside Hubei province thought they were the most likely to be infected, but the residents of cities around Wuhan in Hubei province felt that they were less likely to become infected, and the residents of Wuhan city thought they were the least likely to become infected. Compared to the residents of Wuhan city, the residents of cities around Wuhan in Hubei province and outside Hubei province had the greatest belief in the effectiveness of wearing masks, washing hands and disinfecting, and they were more confident that they could prevent infection in this way. Participants from Wuhan city were the least cautious or most skeptical regarding virus protection measures. The statistical analysis results are shown in Figure 2 below.

### Stress Response Differences Among Residents of the Three Areas

#### Emotional Responses

The ANOVA and LSD shown there are the significant differences in the emotional responses among the residents of the three areas, including being more nervous and anxious [*F*_(2, 4,073)_ = 8.985, *P* < 0.001] afraid for no reason [*F*_(2, 4,073)_ = 12.273, *P* < 0.001], easily upset or frightened [*F*_(2, 4,073)_ = 9.931, *P* < 0.001], depressed [*F*_(2, 4,073)_ = 5.541, *P* = 0.004], and unable to calm down [*F*_(2, 4,073)_ = 7.335, *P* = 0.001]. Residents of cities outside Hubei province were the most nervous and anxious, the most afraid for no reason, the most easily upset or frightened, the most depressed, and the most unable to calm down, while the residents of Wuhan city and cities around Wuhan in Hubei province reported these negative feelings less frequently. The residents of Wuhan city were the least afraid for no reason, and the residents of cities around Wuhan in Hubei province were the least nervous and anxious, least upset or frightened, least depressed, and least unable to calm down.

#### Somatic Reactions

The ANOVA and LSD showed that there was no significant difference in losing weight [*F*_(2, 4,073)_ = 2.58, *P* = 0.076] among the residents of the three areas, Most participants did not significantly lose weight as a result of the epidemic. However, there was a significant difference in the participants' moods and whether they felt tired for no reason [*F*_(2, 4,073)_ = 3.077, *P* = 0.046]. The residents of cities outside Hubei province were the most prone to feel tired for no reason. The residents of Wuhan city and of cities around Wuhan in Hubei province felt comfortable, and the residents of cities around Wuhan in Hubei province were the most comfortable. The statistical analysis results are shown in Figure 3 below.

### Behavioral Response Differences Among the Residents of the Three Areas

#### Attention to Information

The ANOVA and LSD showed that there were significant differences in terms of the participants' attention to authorities [*F*_(2, 4,073)_ = 16.076, *P* < 0.001] and whether they looked for information resources for self-adjustment [*F*_(2, 4,073)_ = 6.005, *P* = 0.002]. The residents of cities outside Hubei province paid attention to authoritative information, but the residents of Wuhan city and of cities around Wuhan in Hubei province were less concerned. The residents of cities outside Hubei province more frequently searched for psychological adjustment information, while the residents of Wuhan city and the residents of cities around Wuhan in Hubei province needed less psychological adjustment.

#### Behavioral Responses

The ANOVA and LSD showed that there were no significant differences among the residents of the three areas in terms of thinking (making some judgments) that all strangers have the novel coronavirus [*F*_(2, 4,073)_ = 0.118, *P* = 0.897], frequently washing or cleaning hands [*F*_(2, 4,073)_ = 2.186, *P* = 0.112], and taking the initiative to avoid strangers [*F*_(2, 4,073)_ = 2.753, *P* = 0.064]. Most participants thought that strangers were carrying the virus, agreed that washing or cleaning hands could prevent the virus and practiced hand washing frequently, and took the initiative to avoid strangers. The data also showed that there was a significant difference in wanting to have a physical investigation [*F*_(2, 4,073)_ = 33.537, *P* < 0.001] among residents of the three areas. The residents of Wuhan city were the most doubtful about their health and more eager to have a general investigation. The residents of cities around Wuhan in Hubei province were less eager to have a general investigation, and the residents of cities outside Hubei province were the most eager. The statistical analysis results are shown in Figure 4 below.

## Discussion

The survey data show proof of a PTE effect at the initial stages of the COVID-19 outbreak (17, 34), but no ripple effect was observed. Specifically, the respondents showed PTE effects in their cognition and emotions (the degree of their cognitive and emotional responses increased along with geographical distance) but did not show PTE effects in their behaviors.

### Psychological Typhoon Eye Effect of Cognition

As the number of confirmed cases and deaths from the epidemic continued to rise daily, most residents truly felt the threat from the virus, and participants in Wuhan, cities around Wuhan in Hubei province, and cities outside Hubei province generally believed that the NCP was horrible (18). In addition, participants from the three regions exhibited some significant differences in other aspects of their cognition of the epidemic, especially in places that differed from the usual perception, clearly showing psychological typhoon eye effects. For instance, residents of cities outside Hubei province thought they were most at risk of contracting COVID-19, and residents of Wuhan city and cities around Wuhan in Hubei province perceive lower risk of contracting COVID-19. Residents of Wuhan city perceived the least danger of the virus spreading and expressed the least support for isolating villages. Residents of Wuhan had the least cognition of protection consciousness and the lowest proportion of participants who scored highly (wearing masks, washing their hands frequently, and disinfecting regularly). Although Wuhan was the worst-hit area, with the highest number of people infected and the highest speed of transmission, among the study participants, the Wuhan residents had the lowest perception of whether they could be infected with the virus.

### Psychological Typhoon Eye Effect of Emotions

The psychological typhoon eye effect was the most obvious in the emotions of residents during the epidemic period, and this was reflected in multifarious negative emotions such as anxious thought, fearful thought, and depressed mood. Participants who were farther from the center of the epidemic had a higher intensity of negative emotions than those who were closer to the center. The proportion of residents in cities outside Hubei province who responded positively to the item “it was easier to be nervous thought and anxious thought than usual” was higher than the proportion of residents of the other two regions who responded the same way. Compared to residents of the other regions, Wuhan residents showed the most stable state of mind; they had the lowest proportion of unprovoked fear. Residents outside Hubei province were the most likely to feel depressed, uneasy, and restless, and the Wuhan participants were the least likely. The residents who were closer to the center of the epidemic had more positive emotions and were more relaxed than the residents of the other two regions.

### Psychological Typhoon Eye Effect of Behaviors

During the transmission of COVID-19, residents were able to consciously take protective measures, such as washing their hands frequently and wearing masks. Regarding behavioral responses, participants were able to diversify their web-based messaging platforms to maximize the breadth and depth of their knowledge about COVID-19. Meanwhile, significant psychological typhoon eye effects were not found in the behavioral responses of residents. Residents outside Wuhan city paid more attention to the epidemic and authoritative information that was released. In addition, compared with the other two regions, residents from outside Hubei province had the highest average score for active self-adjustment; residents outside Hubei province were more inclined to look for information resources to deal with their negative emotions and more actively relieve stress.

The residents across the three areas show consistent behavioral responses to the pandemic, and these responses may have been more stable than their emotions and cognition. Therefore, a sustainable healthcare service for guiding cognition and emotions is urgently needed during the COVID-19 outbreak, and different measures should be adopted across various areas.

### Limitations

This study had several limitations (32). First, the questionnaire was a self-report questionnaire in the context of China's culture, and the psychological symptoms and assessments were not confirmed via clinical evaluations. Second, the residents were just the young group (people) recruited in social media. Third, the study design was cross-sectional, which fails to provide valid information about the previous mental health of the subjects.

## Clinical Implications

The World Health Organization declared the COVID-19 outbreak a pandemic (35), and many countries have introduced social distancing measures (with some cities even closing themselves to public) to curb the transmission of the virus. These measures, which have ranged from mandatory quarantine to voluntary self-isolation, have socially isolated many people, thereby placing their mental and emotional health at risk (36). Based on our findings related to the PTE effect of the pandemic, mental health intervention measures for residents residing in different areas or countries should be an important part of national disaster programs. Governments, media platforms, mental health services, and social support groups can also help alleviate negative cognition and emotions.

### Mental Health Intervention Measures for Residents of the Worst-Hit Areas

#### Strengthening Emotional Guidance

The survey found that compared to residents of the two other regions, residents of Wuhan were more noncommittal about negative states, paid less attention to authoritative and positive events, and appeared to be in a more careless state. This might be related to long-term exposure to a dangerous environment, resulting in taking a dim view. Thus, we should quickly inform those near the center of the epidemic about the seriousness of epidemic prevention and control, strengthen their awareness of the crisis, and place the whole city on alert. In addition, more influential news media, especially the WeChat official account that most people paid attention to, should provide some credible scientific resources about how to correctly understand and view self-emotion and how to effectively adjust negative emotions.

#### Strengthening Education About Protection Consciousness

The survey found that residents of Wuhan had insufficient awareness of virus protection and paid less attention to official information than residents of the other two areas. Therefore, relevant media needs to pay close attention to the situations of residents; do a good job as disseminators of the official “virus protection guide,” popular science, and protection knowledge; improve residents' protection consciousness; enhance risk assessment and prevention awareness; urge residents to objectively and carefully understand the characteristics and dynamics of the epidemic situation; pay attention to the sources of infection risk; prevent residents from being inattentive and blindly optimistic; and encourage residents to develop good health habits.

#### Strengthening Training on Effective Responses

The study found that compared to residents of the two other regions, people in Wuhan were less concerned about authoritative information, were more eager to thoroughly check their physical condition, and were less likely to adopt appropriate channels for psychological adjustment to reduce their stress. Obviously, these actions are not conducive to maintaining psychological balance in the face of an epidemic, and they tend to aggravate people's negative emotions. Different behavioral responses to stressful events can also affect individuals' psychological responses and stress states, especially in the subconscious context. Therefore, we should guide people near the center of the epidemic to acknowledge their objective environments, internal emotions and stress responses and adopt appropriate, reasonable, and effective response behaviors to attempt to solve problems. Furthermore, the government should establish psychological support institutions and strengthen publicity and training to enhance the awareness and behavior of Wuhan citizens seeking psychological assistance.

### Mental Health Intervention Measures for Residents Outside the Worst-Hit Areas

#### Eliminating Unprovoked Fear and Building a Sense of Security

The survey found that residents outside Wuhan were more worried, more uncertain about the risk, more afraid of being infected, and less confident than residents of the other two regions. We need to pay attention to this category of residents and provide emotional counseling (37, 38), reducing tension and improving logical thinking to help them to vent their tension and fear. Relevant information release platforms should guide them to examine whether their access to information is reliable to examine whether the source of their own insecurity and threats is true and to improve their information literacy and reasonable questioning ability to correctly assess the accuracy of information. For areas outside the center of the epidemic, it is suggested that the government should increase information transparency, provide timely disclosure of the latest status of the epidemic, offer timely refutation of rumors, and use simple and easy to inform people about the current status and how to address it.

#### Timely “Disconnection” From Negative Information

The survey found that compared to residents of the other two regions, residents outside Wuhan tended to pay more attention to information from the Internet, and their emotions were more easily affected. Many residents suffered from receiving negative and upsetting information and fell into a vicious cycle of “hypochondriac concerns - physical discomfort - anxiety aggravation”; the symptom characteristics were examined by consideration of an anxious mood, depressed mood, anxiety sensitivity (39). Although information is useful, people should not be too eager to read it, as this can make it difficult to eliminate negative emotions, and residents such as these will be more prone to worry, fear, and other negative emotions. Currently, what is needed is timely “separation” that can allow these individuals to decrease the amount of attention they give to the epidemic situation. The correct reporting principle was neither complete epidemic information nor no epidemic information, which could make residents work and rest regularly.

#### Accurate Cognition of Stress and Acceptance of Self-Response

During an epidemic situation, everyone experiences different degrees of psychological distress, and we should therefore provide early warnings in a timely way so that residents are ready to accept negative emotions and psychological problems. The relevant departments should work to calm and help residents during the epidemic so that they do not experience as much doubt and worry. The relevant departments should also guide residents to try to accept some negative emotions and their own reactions so that they are aware of psychological changes and canf become more accepting of the objective existence of a negative psychological state during the epidemic. This will allow residents to look at and recognize themselves to then solve problems and face psychological crises more rationally.

## Conclusion

The current research indicated that COVID-19 had affected the mental health and daily lives of the residents of three areas. Residents who were closer to the center of the epidemic were more relaxed, less anxious or panicked, paid less attention to authoritative and psychological adjustment information, sensed less danger, and experienced fewer emotional reactions in the earlier phase of COVID-19 than residents who were farther away. More attention should thus be paid to this group.

## Data Availability Statement

The raw data supporting the conclusions of this article will be made available by the authors, without undue reservation.

## Ethics Statement

The study was designed in accordance with the tenets of the Declaration of Helsinki. Approval from the ethical authority of School of Educational Science, Huazhong University of Science and Technology was granted. Confidentiality and the statement confirming informed consent was managed by placing anonymous coding of one self-report questionnaires.

## Author Contributions

YZ, GW, SX, FH, YB, ML, NL, and XC: conceived and designed the questionnaire. GL and JZ: recruitment and payment of participants. GW, YZ, and QX: analyzed the data. GW, SX, GL, and XC: wrote and revised the paper. All authors contributed to the article and approved the submitted version.

## Conflict of Interest

The authors declare that the research was conducted in the absence of any commercial or financial relationships that could be construed as a potential conflict of interest.

## References

[1] Jin Y-H Cai L Cheng Z-S Cheng H Deng T Fan Y-P A rapid advice guideline for the diagnosis and treatment of 2019 novel coronavirus (2019-nCoV) infected pneumonia (standard version). Military Med Res. (2020) 7:4 10.1186/s40779-020-0233-6 PMC7003341 32029004

[2] Sun P Lu X Xu C Sun W Pan B. Understanding of COVID-19 based on current evidence. J Med Virol. (2020) 92:548–51. 10.1002/jmv.25722 32096567 PMC7228250

[3] China Adopts COVID-19 as Official English Name for Disease. Available online at: https://www.chinadaily.com.cn/a/202002/22/WS5e506f18a31012821727970f.html (accessed February 22, 2020).

[4] Harapan H Itoh N Yufika A Winardi W Mudatsir M. Coronavirus disease 2019 (COVID-19): a literature review. J Infect Public Health. (2020) 13:667–73. 10.1016/j.jiph.2020.03.019 32340833 PMC7142680

[5] Zu ZY Jiang MD Xu PP Chen W Zhang LJ. Coronavirus disease 2019 (COVID-19): a perspective from China. Radiology. (2020) 296:E15–25. 10.1148/radiol.2020200490 32083985 PMC7233368

[6] Backer JA Klinkenberg D Wallinga J. Incubation period of 2019 novel coronavirus (2019-nCoV) infections among travellers from Wuhan, China, 20–28 January 2020. Eurosurveillance. (2020) 25:2000062. 10.2807/1560-7917.ES.2020.25.5.2000062 32046819 PMC7014672

[7] Albrecht R Knapp J Theiler L Pietsch U. Transport of COVID-19 and other highly contagious patients by helicopter and fixed-wing air ambulance: a narrative review and experience of the Swiss air rescue Rega. Scand J Trauma Resuscit Emerg Med. (2020) 28:40. 10.1186/s13049-020-00734-9 32410706 PMC7222521

[8] Zhang B Ye T Heng S Levy MZ Small DS. Protocol for an observational study on the effects of social distancing on influenza-like illness and COVID-19. Osong Public Health Res Perspect. (2020) 11:91–2. 10.24171/j.phrp.2020.11.3.07 32494566 PMC7258883

[9] Hui DS Azhar IE Madani TA Ntoumi F Kock R Dar O. The continuing 2019-nCoV epidemic threat of novel coronaviruses to global health—The latest 2019 novel coronavirus outbreak in Wuhan, China. Int J Infect Dis. (2020) 91:264–6. 10.1016/j.ijid.2020.01.009 31953166 PMC7128332

[10] Parr J. Pneumonia in China: lack of information raises concerns among Hong Kong health workers. Br Med J. (2020) 368:m56. 10.1136/bmj.m56 31915179

[11] Allden K Jones L Weissbecker I Wessells M Bolton P Betancourt TS. Mental health and psychosocial support in crisis and conflict: report of the Mental Health Working Group. Prehospital Disast Med. (2009) 24(S2):s217–27. 10.1017/S1049023X00021622 19806544

[12] Cao W Fang L Xiao D. What we have learnt from the SARS epdemics in mainland China? Global Health J. (2019) 3:55–9. 10.1016/j.glohj.2019.09.003 32501415 PMC7148657

[13] Mayhorn CB McLaughlin AC Warning the world of extreme events: a global perspective on risk communication for natural and technological disaster. Saf Sci. (2014) 61:43–50. 10.1016/j.ssci.2012.04.014

[14] Carmo EH Teixeira MG. Technological disasters and public health emergencies: the case of oil spill on the Brazilian coast. Cadernos Saúde Públ. (2020) 36:e00234419. 10.1590/0102-311X00234419 32022178

[15] Xie X-F Stone E Zheng R Zhang R-G The ‘Typhoon Eye Effect’: determinants of distress during the SARS epidemic. J Risk Res. (2011) 14:1091–1107. 10.1080/13669877.2011.571790

[16] Kasperson RE Renn O Slovic P Brown HS Emel J Goble R The social amplification of risk: a conceptual framework. Risk Anal. (1988) 8:177–87. 10.1111/j.1539-6924.1988.tb01168.x

[17] Zhang SX Huang H Wei F. Geographical distance to the epicenter of Covid-19 predicts the burnout of the working population: Ripple effect or typhoon eye effect? Psychiatry Res. (2020) 288:112998. 10.1016/j.psychres.2020.112998 32325386 PMC7194871

[18] Wu W Zhang Y Wang P Zhang L Luo M. Psychological stress of medical staffs during outbreak of COVID and adjustment strategy. J Med Virol. (2020) 93:1962–70. 10.1002/jmv.25914 32314806 PMC7264502

[19] Fangfang W Shuhan M Hanxue Y Yue Q Bin Z “Psychological Typhoon Eye Effect” and “Ripple Effect”: Double perspective test of risk perception and anxiety characteristics of people in different COVID-19 severity regions. Acta Psychol Sin. (2020) 52:1087–104. 10.3724/SP.J.1041.2020.01087

[20] Maderthaner R Guttmann G Swaton E Otway HJ Effect of distance upon risk perception. J Appl Psychol. (1978) 63:380 10.1037/0021-9010.63.3.380

[21] Melber BD Nealey SM Hammersla J Rankin WL Nuclear Power and the Public: Analysis of Collected Survey Research. Seattle, WA: Battelle Human Affairs Research Center (1977).

[22] Lima ML On the influence of risk perception on mental health: living near an incinerator. J Environ Psychol. (2004) 24:71–84. 10.1016/S0272-4944(03)00026-4

[23] Liang H Xue Y. Investigating public health emergency response information system initiatives in China. Int J Med Inform. (2004) 73:675–85. 10.1016/j.ijmedinf.2004.05.010 15325324 PMC7128295

[24] Okeke CU Armour A Post-landfill siting perceptions of nearby residents: a case study of Halton landfill. Appl Geogr. (2000) 20:137–54. 10.1016/S0143-6228(00)00003-5

[25] Li S Rao L-L Bai X-W Zheng R Ren X-P Li J-Z. Progression of the “Psychological Typhoon Eye” and variations since the Wenchuan earthquake. PLoS ONE. (2010) 5:e9727. 10.1371/journal.pone.0009727 20305817 PMC2840028

[26] Zheng R Rao LL Zheng XL Cai C Wei ZH Xuan YH. The more involved in lead-zinc mining risk the less frightened: a psychological typhoon eye perspective. J Environ Psychol. (2015) 44:126–34. 10.1016/j.jenvp.2015.10.002 32287833 PMC7126010

[27] Festinger L A Theory of Cognitive Dissonance, Vol. 2 London: Stanford University Press (1962).

[28] Wiegman O Gutteling JM Risk appraisal and risk communication: some empirical data from the Netherlands reviewed. Basic Appl Soc Psychol. (1995) 16:227–49. 10.1080/01973533.1995.9646111

[29] Wong ND Evidence for psychosocial risk factors and behavioral interventions in cardiovascular disease. Curr Cardiovasc Risk Rep. (2012) 6:528–33. 10.1007/s12170-012-0270-0

[30] Osika W Montgomery SM Dangardt F Währborg P Gan LM Tideman E. Anger, depression and anxiety associated with endothelial function in childhood and adolescence. Arch Dis Childh. (2011) 96:38–43. 10.1136/adc.2008.152777 19822537

[31] Gifford R Mccunn LJ Appraisals of built environments and approaches to building design that promote well-being and healthy behavior (2012) 87–95.

[32] Zhang Y Kong F Wang L Chen H Gao X Tan X Mental health and coping styles of children and adolescent survivors one year after the 2008 Chinese earthquake. Child Youth Serv Rev. (2010) 32:1403–9. 10.1016/j.childyouth.2010.06.009

[33] Battegay M Kuehl R Tschudin-Sutter S Hirsch HH Widmer AF Neher RA. 2019-novel Coronavirus (2019-nCoV): estimating the case fatality rate–a word of caution. Swiss Med Weekly. (2020) 150:w20203. 10.4414/smw.2020.20203 32031234

[34] Zhang Y Cao X Wang P Wang G Lei G Shou Z. Emotional “inflection point” in public health emergencies with the 2019 New Coronavirus Pneumonia (NCP) in China. J Affect Disord. (2020) 276:797–803. 10.1016/j.jad.2020.07.097 32738664 PMC7369017

[35] Qin L Sun Q Wang Y Wu K-F Chen M Shia B-C. Prediction of the Number of New Cases of 2019 Novel Coronavirus (COVID-19) Using a Social Media Search Index. Taiwan: Social Science Electronic Publishing. 10.3390/ijerph17072365 PMC7177617 32244425

[36] Razai MS Oakeshott P Kankam H Galea S Stokes-Lampard H. Mitigating the psychological effects of social isolation during the covid-19 pandemic. BMJ. (2020) 369:m1904. 10.1136/bmj.m1904 32439691

[37] Dawood NA 1 The efficacy of rational-emotional counseling program in reducing tension and improving logical thinking among tenth grade female students. Dirasat. (2001) 28:289–311.

[38] Jiahua WU Feng Y Yuchan LI Galactophore DO Effect of eight methods of emotional counseling on life quality of patients with operable breast cancer during perianticancer chemotherapeutic period. China Med Herald. (2015) 12:134–7.

[39] Otto MW Pollack MH Sachs GS Rosenbaum JF Hypochondriacal concerns, anxiety sensitivity, and panic disorder. J Anxiety Disord. (1992) 6:93–104. 10.1016/0887-6185(92)90008-U

